# Differential expression of microRNAs following cardiopulmonary bypass in children with congenital heart diseases

**DOI:** 10.1186/s12967-017-1213-9

**Published:** 2017-05-30

**Authors:** Masood Abu-Halima, Martin Poryo, Nicole Ludwig, Janine Mark, Ina Marsollek, Christian Giebels, Johannes Petersen, Hans-Joachim Schäfers, Ulrich Grundmann, Thomas Pickardt, Andreas Keller, Eckart Meese, Hashim Abdul-Khaliq

**Affiliations:** 1 Department of Human Genetics, Saarland University, 66421 Homburg/Saar, Germany; 2 Department of Pediatric Cardiology, Saarland University Medical Center, 66421 Homburg/Saar, Germany; 3 Department of Thoracic and Cardiovascular Surgery, Saarland University Medical Center, 66421 Homburg/Saar, Germany; 4 Department of Anaesthesiology, Intensive Care and Pain Therapy, Saarland University Medical Center, 66421 Homburg/Saar, Germany; 5 Competence Network for Congenital Heart Defects, National Register for Congenital Heart Defects, DZHK, 13347 Berlin, Germany; 6 Department of Clinical Bioinformatics, Saarland University, 66041 Saarbruecken, Germany; 7 Department of Human Genetics, Saarland University Medical Center, 66421 Homburg/Saar, Germany

**Keywords:** MicroRNA, Congenital heart disease, Atrial myocardium, Cardiopulmonary bypass

## Abstract

**Background:**

Children with congenital heart defects (CHDs) are at high risk for myocardial failure after operative procedures with cardiopulmonary bypass (CPB). Recent studies suggest that microRNAs (miRNA) are involved in the development of CHDs and myocardial failure. Therefore, the aim of this study was to determine alterations in the miRNA profile in heart tissue after cardiac surgery using CPB.

**Methods:**

In total, 14 tissue samples from right atrium were collected from patients before and after connection of the CPB. SurePrint™ 8 × 60K Human v21 miRNA array and quantitative reverse transcription-polymerase chain reaction (RT-qPCR) were employed to determine the miRNA expression profile from three patients before and after connection of the CPB. Enrichment analyses of altered miRNA expression were predicted using bioinformatic tools.

**Results:**

According to miRNA array, a total of 90 miRNAs were significantly altered including 29 miRNAs with increased and 61 miRNAs with decreased expression after de-connection of CPB (n = 3) compared to before CPB (n = 3). Seven miRNAs had been validated using RT-qPCR in an independent cohort of 11 patients. Enrichment analyses applying the KEGG database displayed the highest correlation for signaling pathways, cellular community, cardiovascular disease and circulatory system.

**Conclusion:**

Our result identified the overall changes of the miRNome in right atrium tissue of patients with CHDs after CPB. The differentially altered miRNAs lay a good foundation for further understanding of the molecular function of changed miRNAs in regulating CHDs and after CPB in particular.

**Electronic supplementary material:**

The online version of this article (doi:10.1186/s12967-017-1213-9) contains supplementary material, which is available to authorized users.

## Background

Congenital heart diseases (CHDs) are the most common congenital organ malformations in newborns [1]. Nearly 10 of 1000 newborns have congenital heart and vessel defects with a wide variation with regards to the severity of defects and the need for immediate therapeutic intervention [1–3]. Advances in diagnostic and therapeutic methods have improved the long-term survival and morbidities of these patients [3–5]. However, considerable mortality rates in neonates rather than older children in association with corrective cardiac surgery remain challenging [1–5]. Neonates needing surgical intervention in early life still have significantly higher mortality than infants and older children [6, 7]. Corrective cardiac surgery is one of the most major cause of mortality in neonates and infants [8]. The morbidities and risk factors associated with cardiopulmonary bypass (CPB) are well documented in adults and children [9–12]. Data on the mechanisms of adaptation and remodeling in the myocardium of neonates and small children undergoing cardiac surgery are still scarce. Therefore, a better understanding of the molecular and genetic mechanisms underlying congenital heart diseases and the specific adapting mechanisms during stress and ischemia is fundamental. Recent studies in adults with different cardiac morbidities have suggested an important role for microRNAs (miRNAs) in the pathogenesis of numerous cardiovascular diseases [13, 14]. MiRNAs are a class of small non-coding RNAs that regulate gene expression post-transcriptionally via sequence-specific interaction with the 3′ UTR of target mRNAs, resulting in mRNA degradation and/or inhibition of translation [15]. In signaling and transcriptional processes of cardiac biology, miRNAs can act as ‘molecular switches’ of post-transcriptional regulation of gene expression by regulating the expression of multiple proteins that function at different steps in cellular processes such as growth, differentiation, metabolism and apoptosis [16] as well as cardiac remodeling including increased angiogenesis and decreased fibrosis [17]. These miRNAs-regulated pathways are contributing to the development of the cardiac remodeling process and their activation following a myocardial injury suggest a functional role for these signaling pathways in cardiac repair. MiRNAs known to be involved in myocardial development include miR-208a, miR-21, miR-145, miR-1, miR-133a and miR-29 [18–20]. Moreover, their aberrant expression has been linked to cardiac remodeling and hypertrophy [18–20]. However, the role of miRNAs in the myocardium under non-physiological conditions of CPB is not well understood. In this study, we aimed to characterize the miRNA profile in the atrial myocardium before and after cardiac surgery by means of CPB in patients (infants and children) with CHDs.

## Methods

### Study population and sample collection

Institutional Review Board approval (No. 156/14) was obtained before initiation of this study. Children aged from 5 days to 10.4 years who had cardiac surgery for CHDs were included in this pilot study after written informed consent was obtained. Selected demographics and preoperative characteristics of the children including diagnoses, surgical procedure and duration of hospitalization are shown in Table 1. Patients were excluded from the study when parents declined participation, complications occurred during surgery or scarring prevented secure extraction of a tissue sample. All patients received midazolam (0.1 mg/kg) for premedication. After preoxygenation with 100% oxygen, anesthesia was induced with thiopental and fentanyl in titrated doses. Atracurium (0.5 mg/kg) was given to facilitate nasotracheal intubation with a cuffed tube. Thereafter anesthesia was maintained with continuous infusions of fentanyl (5–10 µg/kg/h) and midazolam (0.1–0.2 mg/kg/h). The lungs were ventilated with oxygen/air to maintain normocapnia at pH of 7.35–7.45. Volume replacement was done to maintain physiological CVP and arterial pressure. Aortic and atrial cannulation were performed after systemic heparin administration with intravenous bolus. Before cannulation, the CPB was filled with priming solution which consisted of Sterofundin ISO, Gelafundin, Ringer’s lactate and tranexamic acid and then mixed with packed red blood cells (PRBCs) from donors. In every case the Heart–Lung Machine: JOSTRA HL 20 (Maquet, Rastatt, Germany) was used. Blood cardioplegia of 8–10 °C was given according to the Calafiore scheme [21]. Atrial myocardial tissue samples from all patients were collected from the right auricular appendage or the right atrium before cannulation and after de-cannulation of the right atrial cannula. Tissue samples of interest were transferred to siliconized microcentrifuge tubes and immediately frozen in liquid nitrogen. These samples were stored in liquid nitrogen at −180 °C until processing.

**Table 1:** Characteristics of patients included in the study

Patient Nr.	Age at surgery	Gender	Weight (kg)	Diagnosis	Surgical procedure	Duration of CPB (min)	Lowest body temperature (°C)	Duration of hospitalization (days)
1	0.5 year	Male	5.5	ASD II	Closure of the ASD	45	35.1	12
2	0.9 year	Female	8.5	VSD	Closure of the VSD	41	34.4	10
3	10.4 year	Female	33.0	Doubly committed VSD, double chambered right ventricle	Closure of the VSD	82	34.0	10
4	0.5 year	Male	5.2	VSD, ASD II	Closure of the ASD	79	29.4	11
5	2.0 year	Female	11	ASD II	Closure of the ASD	32	34.8	9
6	1.1 year	Male	9.4	ASD I	Closure of the ASD	63	33.9	12
7	1.0 year	Female	6.2	Malalignment-VSD	Closure of the VSD	87	31.8	13
8	5 days	Male	3.3	HLHS	Norwood I, Blalock-Taussig shunt	136	24.2	25
9	7 days	Male	4.1	d-TGA	Switch	112	24.0	24
10	11 days	Male	2.1	TAPVC	Correction of TAPVC	124	22.7	54
11	1.2 year	Male	7.6	ASD II	Closure of the ASD	34	34.5	8
12	1.7 year	Male	10.1	ASD I, mitral insufficiency	Closure of the ASD	41	32.8	8
13	3.5 year	Female	13.0	ASD II	Closure of the ASD	24	34.8	7
14	1.4 year	Female	10.6	ASD II	Closure of the ASD	23	35.0	8

ASD II, Atrial septal defect type II; VSD, membranous ventricular septal defects; HLHS, hypoplastic left heart syndrome; d-TGA, dextro-transposition of the great arteries; TAPVC, total anomalous pulmonary venous connection

### Isolation of total RNA, including miRNAs, from myocardial tissue samples

Myocardial (right atrium) tissue samples were cut into small pieces, crushed and homogenized using the ball mill TissueLyser LT (Qiagen, Hilden, Germany) with an oscillation frequency of 50 Hz. Thereafter, total RNA including miRNA was extracted using the miRNeasy Mini kit on a QIAcube robot (Qiagen, Hilden, Germany) according to the manufacturer’s instructions. Concentration and purity of the samples were measured using the NanoDrop ND-1000 Spectrophotometer (Thermo Fisher Scientific, Massachusetts, USA). RNA integrity was assessed with an Agilent 2100 Bioanalyzer using RNA 6000 Nano kit (Agilent Technologies, California, USA). DNase I (Thermo Fisher Scientific, Massachusetts, USA) treatment was carried out according to the manufacturer’s instructions to remove any DNA contamination. Conventional PCR with exon spanning primers for Glyceraldehyde 3-phosphate dehydrogenase (GAPDH) was performed to exclude residual DNA in the samples [22].

### MiRNA microarray

MiRNA expression profiles of three samples from patients with CHD, before CPB (n = 3) and after CPB (n = 3) were hybridized on a SurePrint G3 miRNA Array v21.0 (Agilent Technologies, California, USA). The patients were chosen based on their age at the time of surgery to largely cover the range of ages included in our study. Briefly, 100 ng input of RNA from each sample was dephosphorylated by incubation with calf intestinal phosphatase and denatured using 100% DMSO. Samples were labeled with pCp-Cy3 by using T4 ligase. Each labeled RNA sample was hybridized onto an individual 8 × 60K format Agilent miRNA array slide v21. Arrays were washed and dried according to manufacturer’s recommendations and scanned at a resolution of 3 μm double-pass mode using an Agilent scanner. Data were acquired using Agilent AGW Feature Extraction software version 10.10.11 (Agilent Technologies, California, USA).

### Reverse transcription qPCR of miRNA (array validation)

To validate the microarray results in the study, relative quantitative real-time PCR was performed on an ABI StepOnePlus™ Real-Time PCR System (Applied Biosystems, Foster City, USA) using SYBR Green I on nine differentially expressed miRNAs. Complementary DNA (cDNA) was generated by reverse transcription of 200 ng of total RNA using miScript RT II Kit (Qiagen, Hilden, Germany). Briefly, 200 ng of total RNA containing miRNAs was mixed with miScript 4 µL HiSpec Buffer, 2 µL nucleic mix, 2 µL miScript Reverse Transcriptase mix and RNase-free water to a final volume of 20 µL. Following the reverse transcription reaction, the cDNA was diluted 1:10 and then mixed with 10 µL QuantiTect SYBR Green PCR Master Mix, 2 µL miScript Universal Primer, 2 µL miScript Primer Assay for the selected nine miRNAs namely miR-328-5p, miR-4750-5p, miR-210-5p, miR-423-3p, miR-143-3p, miR-564, miR-770-5p, miR-874-5p, miR-93-5p and RNU6B as endogenous control and RNase-free water to a final volume of 20 µL. The primer sequences used in the study are shown in Additional file 1: Table S1. Reactions were assembled with the QIAgility automated pipetting system (Qiagen, Hilden, Germany). All PCR data were analyzed with SDS Relative Quantification Software version 2.3 (Applied Biosystems, Foster City, USA).

### Target prediction and functional analysis

Enriched KEGG pathway analyses were performed using DIANA-miRPath v.3.0 software based on predicted targets by DIANA-microT-CDS [23]. Targets of miRNAs with a score of more than 0.8 were selected. Only KEGG pathways with* P* value <0.05 and a false discovery rate (FDR) <0.05 were retained. The effect of miRNAs on target genes and networks has been evaluated using miRTargetLink software [24].

### Reverse transcription qPCR of miRNA and mRNA (functional network validation)

We used miScript Primer Assays for 9 miRNAs (miR-744-5p, miR-648, miR-193b-3p, miR-212-3p, miR-143-3p, miR-93-5p, miR-222-3p, miR-423-3p and miR-766-3p) and QuantiTect Primer assays for 9 target genes (CDKN1A, MYC, PTEN, ESR1, ETS1, SOD2, MGMT, KRAS and HNF4A) (Qiagen, Hilden, Germany) to validate the different expression levels of the miRNA and their target genes, which are determined by miRTargetLink prediction software. In brief, 350 ng of total RNA including miRNA were converted into cDNA using the *mi*Script II RT Kit. During the reverse transcription step, 5× miScript HiFlex Buffer was used to promote conversion of RNA into cDNA. The resulting cDNA was diluted to obtain a miRNA concentration of 1.5 ng/µL and a mRNA concentration of 5 ng/µL for. All reverse transcription PCR (RT-PCR) experiments were performed using the QIAgility™ automated PCR setup (Qiagen, Hilden, Germany). RT-qPCR analysis was done on a StepOnePlus™ Real-Time PCR system (Applied Biosystems, Foster City, CA, USA). GAPDH and RNU6B were chosen as reference genes for mRNA and miRNA normalization, respectively. In addition, we included a no-template control (NTC) and no-reverse transcriptase control (NRT) in each run.

### Statistical analysis

The freely available R software (R Development Core Team, 2010) was used to analyze the differences in miRNA expression in the atrial myocardial tissue samples from patients with CHD samples before and after CPB. After applying the Agilent Feature Extraction image analysis software on our hybridized microarray slides, we collected the computed total gene signals (TGS) for each miRNA, and performed quantile normalization and a log (base 2) transformation of the TGS values. Differential levels of miRNAs were analyzed by employing a paired two-tailed t-test for miRNAs that showed a significant change in the two groups. * P*-values below a threshold of 0.05 were considered statistically significant. We used the relative quantitative method of 2^−ΔΔCq^ to measure the expression differences of specifically selected miRNAs in RT-qPCR [25]. Two-tailed paired T-test was used to evaluate the fold change of miRNA levels.

## Results

### Patient characteristics

A total of 14 patients who had cardiac surgery for CHDs were included in the present study. Preoperative characteristics of the children including diagnoses, surgical procedure and duration of hospitalization are shown in Table 1.

### Differentially expressed miRNAs

**Fig. 1:**
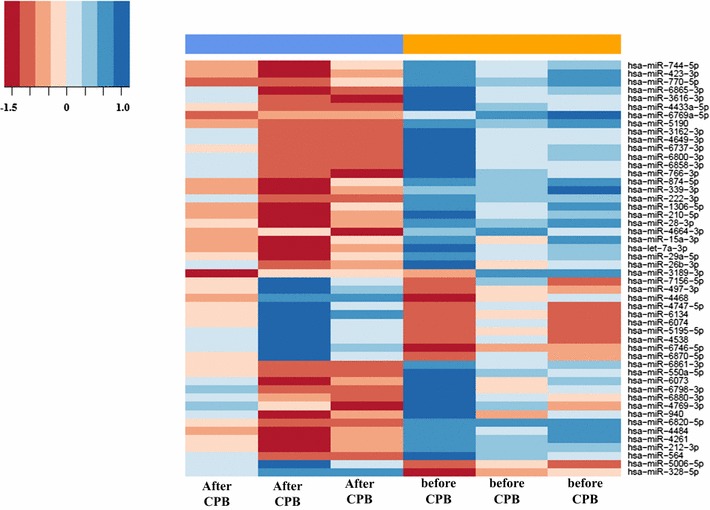
Unsupervised hierarchical clustering (Euclidian distance, complete linkage) of the three patients (six samples, three before and three after CPB) based on expression of the 50 with highest variance. The heatmap shows miRNAs with high expression in blue, miRNAs with low expression in *red*. The *blue* and *orange lines* indicate the two main clusters of samples

In order to characterize miRNA expression profiles in the atrial myocardial tissue of patient after CPB, we performed microarray assays using SurePrint G3 miRNA arrays that contain 2549 human miRNAs annotated in miRBase version 21.0. Atrial myocardial tissue from patients with CHD before cardiac surgery by means of CPB and after CPB were collected (three patients in total). By applying a paired two-tailed t-test, microarray assays showed that miRNAs were expressed differentially in atrial myocardial tissue after surgical treatment with CPB. A total of 90 miRNAs were significantly altered in the two considered groups with a * P* value of <0.05. Out of the 90 altered miRNAs, 29 were up-regulated in the samples after CPB compared to samples before CPB, while 61 miRNA were down-regulated (Table 2). As shown in Table 2, the majority of altered miRNA (49 out of 90) fell into the range of 1.50–1.99 fold up- or down-regulation. In addition, 17 miRNAs including six up-regulated and another eleven down-regulated miRNAs displayed expression level with changes ≥2.0-fold and 24 miRNA including eight up-regulated and another sixteen down-regulated miRNAs displayed expression level with changes <1.5 fold between two groups. Using hierarchic clustering with the euclidian distance measure, we analyzed how the expression profiles of patients before and after CPB relate to each other. For this task, we used the 50 miRNAs with the highest variance of miRNA levels out of the 2.549 miRNAs. Figure 1 shows the heatmap of the hierarchic clustering from the differentially expressed miRNA by microarray. In general, the hierarchical clustering exhibited a clear separation of the examined groups based on miRNA expression profiles. The first cluster contains mostly patients before CPB and the second most of the patients after CPB.

**Table 2:** Significantly expressed miRNAs in the atrial myocardial tissue of patients with CHD after CPB (n = 3) compared to before CPB (n = 3) as determined by microarray

miRNA	Median before CPB	Median after CPB	STDV before CPB	STDV after CPB	Fold change	Regulation	* P*-value
miR-4750-5p	1.67	8.60	2.59	4.24	5.26	Up	0.0399
miR-6134	1.00	3.08	0.46	0.78	3.03	Up	0.0215
miR-6873-3p	2.49	6.29	0.29	1.31	2.50	Up	0.0441
miR-4747-5p	1.07	2.38	0.78	1.05	2.22	Up	0.0151
miR-5195-5p	1.48	3.04	0.49	0.91	2.04	Up	0.0213
miR-6074	1.50	3.00	1.14	1.52	2.00	Up	0.0252
miR-6751-3p	2.89	5.68	0.33	1.12	1.96	Up	0.0323
miR-328-5p	15.50	28.90	4.82	3.56	1.85	Up	0.0263
miR-6792-5p	2.42	4.40	0.56	1.34	1.82	Up	0.0485
miR-4514	1.65	2.93	0.80	0.61	1.79	Up	0.0333
miR-4538	3.31	5.83	1.60	1.37	1.75	Up	0.0033
miR-6870-5p	2.31	3.91	1.63	1.88	1.69	Up	0.0102
miR-7156-5p	1.25	2.12	0.61	0.61	1.69	Up	0.0300
miR-4447	1.64	2.73	0.44	0.19	1.67	Up	0.0369
miR-6769b-5p	20.56	33.29	3.95	4.51	1.61	Up	0.0442
miR-7846-3p	5.11	8.12	1.69	2.65	1.59	Up	0.0398
miR-1261	1.90	2.98	1.58	2.01	1.56	Up	0.0353
miR-6740-5p	39.53	61.77	6.38	14.38	1.56	Up	0.0489
miR-6746-5p	3.75	5.79	1.03	1.20	1.54	Up	0.0265
miR-645	1.56	2.42	0.93	0.70	1.54	Up	0.0455
miR-2113	1.46	2.20	0.47	0.74	1.52	Up	0.0437
miR-497-3p	1.46	2.14	0.29	0.51	1.47	Up	0.0280
miR-648	1.64	2.39	0.55	0.44	1.47	Up	0.0497
miR-5088-5p	9.24	13.26	1.59	2.19	1.43	Up	0.0379
miR-3945	3.43	4.81	0.97	0.98	1.41	Up	0.0342
miR-4468	2.07	2.84	0.55	0.62	1.37	Up	0.0289
miR-1471	6.69	9.07	2.16	2.11	1.35	Up	0.0464
miR-5006-5p	21.99	28.90	2.45	3.83	1.32	Up	0.0108
miR-198	3.59	4.49	1.04	1.32	1.25	Up	0.0404
miR-770-5p	9.59	3.17	1.11	1.81	3.03	Down	0.0095
miR-4261	35.83	14.45	1.98	7.10	2.48	Down	0.0183
miR-874-5p	15.30	6.57	1.76	3.12	2.33	Down	0.0102
miR-550a-5p	5.57	2.44	1.67	1.10	2.29	Down	0.0114
miR-3651	53.15	23.24	5.23	9.24	2.29	Down	0.0455
miR-6865-3p	8.00	3.55	1.51	1.76	2.26	Down	0.0108
miR-222-3p	14.15	6.51	1.03	2.80	2.17	Down	0.0308
miR-3607-3p	8.69	4.10	0.70	1.25	2.12	Down	0.0424
miR-1304-3p	9.97	4.71	1.98	0.36	2.12	Down	0.0469
miR-6508-5p	9.27	4.41	2.31	1.58	2.10	Down	0.0358
miR-6800-3p	10.18	5.06	2.12	2.07	2.01	Down	0.0182
miR-6861-3p	6.13	3.09	0.74	0.86	1.99	Down	0.0139
miR-212-3p	25.83	13.01	2.76	6.93	1.99	Down	0.0294
miR-6737-3p	11.31	5.76	2.96	1.67	1.96	Down	0.0293
miR-6820-5p	37.19	19.53	0.20	4.42	1.90	Down	0.0238
miR-6792-3p	6.60	3.47	1.06	1.47	1.90	Down	0.0366
miR-3616-3p	6.92	3.68	1.31	1.75	1.88	Down	0.0082
miR-3162-3p	10.75	5.79	2.89	2.53	1.86	Down	0.0029
miR-423-3p	9.21	4.94	1.09	1.21	1.86	Down	0.0056
miR-5190	10.43	5.68	0.59	0.86	1.84	Down	0.0172
miR-4725-5p	8.95	4.90	1.35	2.34	1.83	Down	0.0421
miR-744-5p	9.67	5.32	1.56	1.98	1.82	Down	0.0308
miR-6829-5p	48.68	26.75	3.55	8.52	1.82	Down	0.0360
miR-6889-3p	8.18	4.51	1.73	1.02	1.82	Down	0.0432
miR-4433a-5p	8.38	4.62	2.04	1.02	1.81	Down	0.0276
miR-6813-3p	7.19	4.06	2.29	1.01	1.77	Down	0.0343
miR-4649-3p	9.50	5.47	3.12	2.42	1.74	Down	0.0093
miR-6763-3p	7.94	4.62	1.52	1.52	1.72	Down	0.0434
miR-532-3p	18.58	10.78	0.43	3.99	1.72	Down	0.0487
miR-6769a-5p	11.01	6.45	1.97	1.02	1.71	Down	0.0219
miR-193b-3p	74.91	43.92	26.81	34.56	1.71	Down	0.0341
miR-331-5p	2.64	1.58	0.28	0.62	1.67	Down	0.0446
miR-6858-3p	8.53	5.15	2.59	2.47	1.66	Down	0.0017
miR-564	17.68	10.75	2.27	3.32	1.65	Down	0.0099
miR-6723-5p	26.08	16.14	2.83	6.35	1.62	Down	0.0352
miR-28-3p	4.85	3.02	0.21	0.58	1.61	Down	0.0187
miR-339-3p	13.22	8.22	1.98	2.36	1.61	Down	0.0275
miR-23c	5.83	3.62	1.59	0.81	1.61	Down	0.0378
miR-93-5p	114.26	70.98	9.72	24.81	1.61	Down	0.0488
miR-6752-3p	5.76	3.67	1.11	0.79	1.57	Down	0.0329
miR-766-3p	10.39	6.64	1.38	2.07	1.56	Down	0.0125
miR-1306-5p	4.68	3.05	0.44	0.67	1.54	Down	0.0074
miR-6798-3p	4.49	2.92	0.87	1.04	1.54	Down	0.0141
miR-664a-3p	17.21	11.20	1.39	3.72	1.54	Down	0.0362
miR-208a-5p	45.35	29.37	7.47	13.45	1.54	Down	0.0450
miR-6880-3p	6.55	4.43	2.43	1.74	1.48	Down	0.0272
miR-143-3p	327.88	223.74	43.81	69.68	1.47	Down	0.0425
miR-624-5p	3.66	2.49	0.37	0.53	1.47	Down	0.0447
miR-210-5p	4.05	2.86	0.41	0.39	1.42	Down	0.0101
miR-6073	5.18	3.67	1.17	1.43	1.41	Down	0.0140
miR-374c-5p	11.99	8.54	1.55	2.75	1.40	Down	0.0378
miR-4484	27.73	20.62	1.81	1.80	1.34	Down	0.0000
miR-4664-3p	5.01	3.75	0.50	0.61	1.34	Down	0.0096
miR-6731-3p	4.38	3.26	1.21	1.48	1.34	Down	0.0399
miR-4769-3p	6.46	5.11	1.50	1.35	1.26	Down	0.0213
miR-15a-3p	3.49	2.79	0.34	0.28	1.25	Down	0.0114
hsa-let-7a-3p	3.24	2.59	0.35	0.29	1.25	Down	0.0172
miR-26b-3p	3.15	2.54	0.72	0.55	1.24	Down	0.0244
miR-940	32.01	26.08	7.78	6.20	1.23	Down	0.0218
miR-3189-3p	3.61	3.01	0.44	0.33	1.20	Down	0.0153
miR-29a-5p	3.05	2.53	0.16	0.28	1.20	Down	0.0166

Each value represents the median of three patients before and after CBP and ±standard deviation (STDV). Statistical analysis was performed with paired-two-tailed t-test (*P* < 0.05)

### Validation of differentially expressed miRNAs using RT-qPCR

**Fig. 2:**
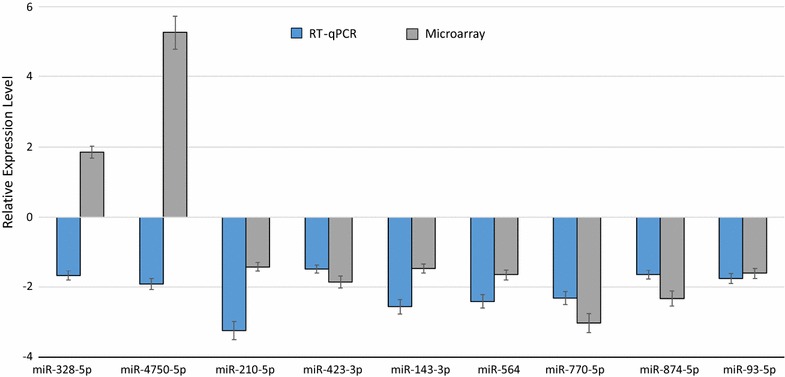
Relative fold change of the miRNAs in the atrial myocardial tissue of patients with CHD after CBP (n = 11) compared to before CPB (n = 11) as determined by RT-qPCR and after CBP (n = 3) compared to before CPB (n = 3) as determined by microarray (*P* < 0.05). Paired-two-tailed t-tests and ±standard deviation (STDV) were used to evaluate differences in expression in both assays. Relative expression level of 2^−ΔΔCt^ and quantile normalization were used for RT-qPCR and microarray data, respectively

**Fig. 3:**
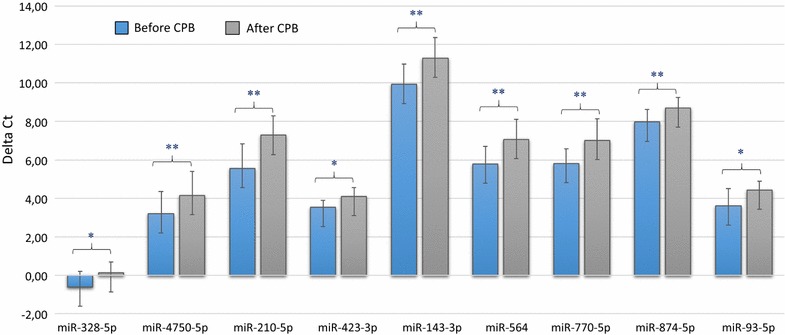
Validation of nine differentially expressed miRNAs in the atrial myocardial tissue of patients with CHD after CBP (n = 11) compared to before CPB (n = 11) as determined by RT-qPCR (*P* < 0.05). Mean ΔCt before CPB and after CPB (Lower ΔCt, higher expression level). RNAU6B as an endogenous control for normalization, paired-two-tailed t-tests and ±standard deviation (STDV) were used to evaluate differences in expression

**Fig. 4:**
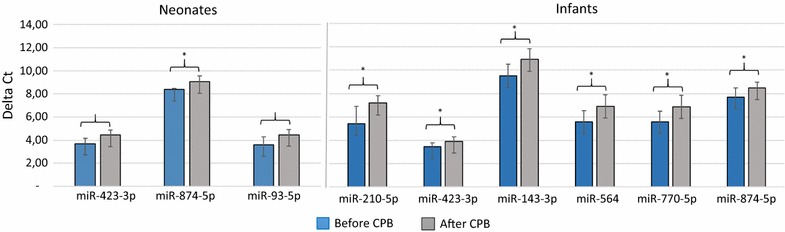
Significantly expressed miRNAs in the atrial myocardial tissue of neonates and infants with CHD after CBP compared to before CPB as determined by RT-qPCR (*P* < 0.05). Mean ΔCt before CPB and after CPB (Lower ΔCt, higher expression level). RNAU6B as an endogenous control for normalization, paired-two-tailed t-tests and ±standard deviation (STDV) were used to evaluate differences in expression

To validate the data obtained from the miRNA microarray, RT-qPCR was performed to re-examine the expression level of nine miRNAs, namely miR-328-5p, miR-4750-5p, miR-210-5p, miR-423-3p, miR-143-3p, miR-564, miR-770-5p, miR-874-5p and miR-93-5p. These miRNAs were chosen based on their differential expression level in each patient group before and after CPB and/or on their known associations with heart diseases. In detail, we selected two miRNAs with highest (miR-4750-5p) and moderate (miR-328-5p) fold change among the up-regulated miRNAs and two miRNAs with highest (miR-770-5p) and moderate (miR-564) fold change among the down-regulated miRNAs. In addition, we selected six miRNAs (miR-328-5p, miR-210-5p, miR-423-3p, miR-143-3p, miR-874-5p and miR-93-5p) with low or moderate expression levels based on the array analysis and with known association with cardiac pathologies and its related process [26–33]. For this validation step, we analyzed a new cohort of 11 samples taken from patients with CHD undergoing corrective surgery by CPB. The RT-qPCR showed the same direction of expression changes as the microarray analysis for seven miRNAs (Fig. 2). The significance of the differences in the expression was confirmed for seven miRNAs namely miR-210-5p, miR-423-3p, miR-143-3p, miR-564, miR-770-5p, miR-874-5p and miR-93-5p) (*P* < 0.05) in the atrial myocardial samples from patients with CHDs after CPB compared to before CPB by RT-qPCR (Fig. 3). By including two out of the seven validated miRNAs namely miR-143-3p and miR-93-5p, a clear distinction between the groups based on the clustering dendrogram was, however, not possible (Additional file 2: Figure S1). We next analyzed the expression levels of the nine miRNAs before CPB and after CPB separately for neonate and infant patients. As for the atrial myocardial samples from neonates with CHDs, we found a significantly different expression for miR-874-5p with a * P*-value of 0.040 between prior CPB and after CPB. In addition, we found two miRNAs with border-line * P*-values (miR-423-3p, * P*-value = 0.060 and miR-93-5p, * P*-value = 0.070) (Fig. 4). As for atrial myocardial samples from infants with CHDs, we found significantly different expression of six miRNAs (miR-210-5p with a * P*-value of 0.014, miR-423-3p with a * P*-value of 0.034, miR-143-3p with a * P*-value of 0.018, miR-564 with a * P*-value of 0.024, miR-770-5p with a * P*-value of 0.025, and miR-874-5p with a * P*-value of 0.040) between prior and after CPB (Fig. 4). In addition, we analyzed the relative expression level of different miRNAs depending on the age of each patient before and after CPB. As shown in Additional file 3: Figure S2, the relative expression level (2^−ΔΔCt^) for certain miRNAs decreased with age. In addition, we found a remarkable difference between the miRNAs analyzed.

### Comparative pathway analysis

**Fig. 5:**
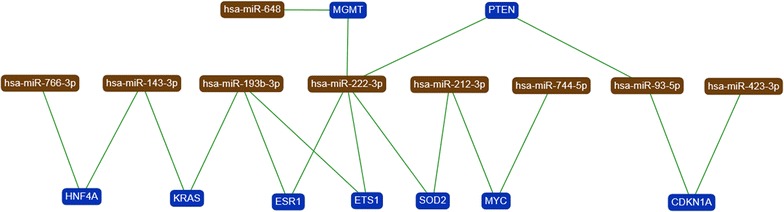
Target network with deregulated miRNAs indicated in *brown* and target genes in *blue*. Only genes targeted by at least two or ore miRNAs are included. Strong interaction was observed between miRNAs and genes are highlighted by “*green*” *edges* in the resulting network

We used DIANA-mirPath algorithm to gain insights into the biological pathways of the miRNAs that were altered in the atrial myocardial tissue from patients with CHD after CPB compared to before CPB. Based on the deregulated miRNAs by microarray analysis, we identified seventy KEGG pathways that were significantly enriched (*P* < 0.05, FDR corrected) for targets of the deregulated miRNAs, respectively (Additional file 1: Table S2). As shown in Table 3, the target genes of the altered miRNAs are mostly involved in “Signaling transduction”, “Cellular community”, “Cardiovascular disease”, “Circulatory system” and many others pathways. Using miRTargetLink, the resulting network is drawn schematically in Fig. 5. Besides the nine miRNAs, nine genes were targeted by one, two or more of the selected miRNAs in the “Strong” category. A strong interaction was observed between miRNAs and genes are highlighted in ‘‘Green’’ in the resulting network (Fig. 5).

**Table 3:** The KEGG pathways significantly enriched for target genes of deregulated miRNAs in the atrial myocardial tissue from patients with CHD after CPB (n = 3) compared to before CPB (n = 3) (* P* value < 0.05)

Mode of interaction	KEGG pathway	* P*-value	No. genes	No. miRNAs
Signaling transduction	Hippo signaling pathway	1.11E + 05	114	70
	Phosphatidylinositol signaling system	1.23E + 06	66	63
	TGF-beta signaling pathway	3.29E + 06	64	53
	cGMP-PKG signaling pathway	3.29E + 06	128	74
	FoxO signaling pathway	0.001	98	72
	ErbB signaling pathway	0.0027	67	62
	MAPK signaling pathway	0.003	179	77
	Ras signaling pathway	0.0035	155	76
	Sphingolipid signaling pathway	0.0145	85	64
	Calcium signaling pathway	0.0174	124	71
	AMPK signaling pathway	0.0189	86	67
	cAMP signaling pathway	0.019	137	71
	TNF signaling pathway	0.0229	76	63
	Rap1 signaling pathway	0.0243	143	70
Cardiovascular diseases and Circulatory system	Adrenergic signaling in cardiomyocytes	1.20E + 06	112	71
	Dilated cardiomyopathy	0.0154	65	57
	Arrhythmogenic right ventricular cardiomyopathy (ARVC)	0.0174	53	54
	Vascular smooth muscle contraction	0.0196	83	65
Cellular community	Adherens junction	3.20E − 03	56	58
	Focal adhesion	0.0044	146	74
	Gap junction	0.0106	65	61
	Cell adhesion molecules (CAMs)	0.0248	98	68

### Validation of miRNAs and mRNAs that were differentially expressed in atrial myocardial tissues

To confirm the differential expression of miRNAs and their targeted mRNAs before and after CPB, we analyzed eight of the down-regulated miRNAs, one of the up-regulated miRNAs and nine target mRNAs by RT-qPCR. With the exception of miR-222-3p, the remaining eight miRNAs showed the same direction of expression changes in the RT-PCR and in the microarray analysis. These findings are summarized in Additional file 4: Figure S3. Out of the eight miRNAs, miR-648 was up-regulated and the seven remaining miRNAs were down regulated (miR-744-5p, miR-193b-3p, miR-212-3p, miR-143-3p, miR-93-5p, miR-423-3p and miR-766-3p). As for the mRNA analysis, 6 out of 9 analyzed target mRNAs showed the expected expression changes. In detail, for five down-regulated miRNAs the according mRNA targets (ESR1, ETS1, MYC, PTEN and CDKN1A) were up-regulated. For the up-regulated miRNA miR-222-3p the according mRNA target (MGMT) was down-regulated. Due to low number of samples, we could not determine the significance of the expression differences. For the targets SOD2 and KRAS we did not find expression changes between prior CPB and after CPB. For the target HNF4A, we could not determine the fold changes.

## Discussion

In our study, we found evidence for an overall altered miRNA expression pattern in the atrial myocardial tissue of patients with CHD according to corrective cardiac surgery by using the cardiopulmonary bypass (CPB). MiRNA expression analysis using microarrays indicated 90 miRNAs with significantly differential expression, including 29 miRNAs with significantly up- and 61 miRNAs with significantly down-regulated expression after CPB. In the validation phase, by using a new cohort of samples, seven miRNAs were validated. These data show that miRNA expression levels in atrial myocardial tissue changes in the course of cardiac surgery using the CPB. MiRNAs specifically expressed or enriched in smooth, skeletal, and/or cardiac muscle that plays a physiological role in normal heart development and function are termed Myo-miRs [34]. In particular, miR-208, a member of the miR-208 family, is differentially expressed during heart developmental and plays an essential role in normal cardiac conduction [34, 35]. Both over-expression and under-expression of miR-208a has been associated with cardiac arrhythmia [36], with apoptosis in ischemic cardiomyocytes [37] and generally with heart diseases [38]. Down-regulation of miR-208a in atrial myocardial tissues of patients with CHD after CPB underscore the integral function of this miRNA in regulating cell morphology and contractility. The cardiomyogenesis regulator miR-143 is highly expressed during vascular smooth muscle cell differentiation and is involved in normal function and formation of the cardiac chamber via regulation of myocardial cell morphology [39]. The down-regulation of miR-143 causes ventricular collapse by affecting cell morphology and contractility [39]. Furthermore, miR-143 targets ETS Transcription Factor (Elk1), an activator of vascular smooth muscle cells proliferation [40].

Deregulation of a number of miRNAs in our study have been previously reported in right and left atrial appendages of patients with rheumatic mitral valve disease (RMVD), including miR-222-3p, miR-4484 and miR-940 in left atrial appendage [41], miR-5190 and miR-23c in right atrial appendages [42] and miR-143-3p in both, in the LAA and RAA of patients with RMVD [43]. MiR-222 has a documented function in regulating cell proliferation and is involved in the vascular smooth muscle cells differentiation [38, 44]. In neonatal cardiomyocytes, miR-222 induces cellular hypertrophy and proliferation and inhibits apoptosis after ischemic injury [45]. In addition, miR-222 increases cell proliferation and inhibits cardiomyogenic differentiation in right ventricular outflow tract (RVOT) myocardial tissues from infants with nonsyndromic tetralogy of fallot (TOF) [46]. Our finding for miR-222 also suggests that this miRNA protects cardiac structure and functions after corrective surgery by CPB. Previous studies have identified that many miRNAs are expressed at low levels under normal condition and expressed strongly during pathological stress like miR-212, a cardiomyocyte-specific miRNA, which is strongly activated during heart failure. Its up-regulation expression leads to pathological cardiac hypertrophy [47]. In agreement with our expression direction, Zhou et al. [48] reported down-regulation of miR-423-3p and miR-532-3p in ischemia–reperfusion injury heart grafts. MiR-423-5p was suggested as a diagnostic biomarker to distinguish heart failure patients from healthy controls. The plasma concentration patients with heart failure may reflect the severity of dilated cardiomyopathy [49]. Similarly, reduced expression of miR-93 was observed in the left atrium compared to right atrial tissue of patients with sinus rhythm [50] indicating that miR-93 promotes angiogenesis in the ischemic tissue by coordinating the functional pathways of cell proliferation and apoptosis [51]. The up-regulation of miR-328 expression suppresses its target gene SERCA2a (ATPase Sarcoplasmic/Endoplasmic Reticulum Ca2+ Transporting 2) in cardiac myocytes to indirectly activate the calcineurin/NFATc3 signaling pathway, leading to cardiac hypertrophy [33]. In addition, miR-328 was up-regulated in the atria of patients with atrial fibrillation (AF) [52]. Two miRNAs, miR-624 and miR-339, were deregulated in ischemic heart disease and coronary artery disease [53, 54]. MiR-744 was deregulated in patients with chronic congestive heart failure [55]. Several miRNAs were detected in cardiac tissue at different stages of development and are highly expressed in the fetal heart, including miR-212, miR-210 and miR-423 [41]. Moreover, Huang et al. found that miR-210 is involved in cellular hypoxia, regulation of angiogenesis and apoptosis [36]. Similarly, miR-423-5p, miR-193b-3p, and miR-550a-5p showed an altered expression level in patients with heart failure [56, 57].

Bioinformatics analysis by DIANA-mirPath predicted several KEGG biological pathways that were significantly enriched for the differentially deregulated miRNAs in the atrial myocardial samples (Table 3). Enrichment analysis displayed the highest correlation for signaling pathways, providing further evidence that myocardial target proteins are involved in the signaling pathways. Pathways related to the cardiovascular system and vascular smooth muscle contraction and its related processes, including cardiomyocytes proliferation, differentiation and apoptosis have been identified [58–63]. Recent studies implicating the Hippo signaling pathway promotes cardiomyocyte proliferation by activating the insulin-like growth factor [64] and Wnt signaling pathways [65]. Similarly, ErbB signaling pathway plays an important role in proper heart morphogenesis and also in all developmental stages of the heart [66]. These results demonstrate that the deregulated miRNAs in atrial myocardial tissue play an important role in participating in many signaling pathways that control heart function. We employed bioinformatics tools to gain further insights into the impact of the deregulated miRNAs on target genes. As indicated in Fig. 5 and Additional file 4: Figure S3 specific genes are likely affected by the several deregulated miRNAs including the genes for O-6-methylguanine-DNA methyltransferase (MGMT) and superoxide dismutase 2 (SOD2). The expression of these genes is enhanced in myocardial remodeling, cardiac hypertrophy and/or failure indicating that severe stress effects plays a critical role in in the pathogenesis of myocardial remodeling and failure [67, 68]. Phosphatase and tensin homolog (PTEN) plays a role in promoting cardiomyocyte proliferation and regeneration and protect the heart from hypertrophy and heart failure under biomechanical stress [69–71]. The transcription factors v-Myc Avin myelocytomatosis viral oncogene homologue (c-Myc) also play a regulatory role in stimulating cardiac myocytes proliferation and differentiation during fetal development [72].

We would like to point out that this study has also a number of limitations. One important point is that the study population was small and heterogeneous; age at surgery ranged from 5 days of life to 10.4 years and we did not differ between male and female gender. Moreover, the examined CHDs reached from simple ASDs to complex CHDs like HLHS or TAPVC and complicated the interpretation of the data. In fact, we were able to find several miRNAs which were elevated in all patients despite the heterogeneous study population. Another limitation is that all samples were taken from the right atrium. Acquisition of the samples happened before cannulation and after de-cannulation. However, for example, it is not clear which impact the suture before excision of the sample on the miRNA expression has. A sampling of a myocardial specimen from the right or left ventricle before the connection to CPB is not possible. This can be done during the corrective surgery after the cardiac arrest and may not represent the physiological conditions before the connection to CPB and cardiac arrest. Thus, the tissue sampling from the atrial appendage may facilitate the examining of the myocardial conditions before and after surgery using the CPB without increasing risks. In addition, this side of the heart may represent nearly a uniform myocardial region in all patients with CHD. Other settings of myocardial sampling will not be approved by the ethic committee of our institution. The precise mechanisms underlying the expressions of such miRNA should be evaluated in a larger cohort of patients including subgroups of uniform diagnosis of congenital heart defects. Nevertheless, we conclude that the connection to CPB and hypothermic cardiac arrest may induce several physiological responses in the myocardium, which in part is reflected by changed miRNA expression levels. Furthermore, it is unclear which effect the cardiac surgery itself as well as the usage of the CPB has on the miRNA expression. Several non-physiological circumstances occur during this process; for example, cardioplegia induces cardiac arrest, hypothermia is used, the heart is mechanical unloaded and reperfusion occurs after CPB. In addition, the CPB means contact of the blood with a foreign body surface. Whether this circumstance alone already causes changes in the miRNA profile has to be cleared.

## Conclusion

In summary, we report miRNAs in the atrial myocardial tissue with significantly altered expression levels in patients with CHD after cardiac surgery with CPB. These altered miRNAs include cardiac-specific miRNAs which have been described in various cardiac pathologies including congenital heart disease. The overlap between the miRNAs identified in our study and the miRNAs that are involved in various levels of cardiac development by either up- or down-regulation strongly supports the idea that these miRNAs play an essential role in the pathophysiology of congenital heart diseases. Although it is important to bear in mind, that the miRNA expression profiles were obtained from a small number of atrial myocardial tissue samples, the altered expression levels have been further confirmed RT-qPCR for nine deregulated in a cohort of 11 independent atrial myocardial tissue samples. The alteration of the expression of miRNAs may provide new insights into the underlying mechanisms of cardiac surgery with CPB and provide potential novel mechanism-based therapeutic strategies for CHD.

## Additional files

**Additional file 1: Table S1.** RT-qPCR primer sequences used in the study. **Table S2.** MiRNA associated pathways in the atrial myocardial tissue of patients with CHD after CPB (n=3) compared to before CPB (n=3): KEGG Pathways with predicted interaction enrichments for deregulated miRNAs by microarray (P value < 0.05).

**Additional file 2: Figure S1.** Unsupervised hierarchical clustering (Euclidian distance, complete linkage) of 3 patients with 6 samples including 3 samples before and 3 samples after CPB. The clustering was done based on the expression of the highest variance miRNAs as determined by RT-qPCR. MiRNAs with high expression are shown in blue and miRNAs with low expression in red. The blue and orange lines indicate the two main clusters of samples.

**Additional file 3: Figure S2.** Influence of age on the expression of 9 differentially expressed miRNA as determined by RT-qPCR. The relative expression level of 2^-ΔΔCt^ is indicated on the y-axis and the patient age at the time of surgery on the x-axis. The different miRNAs are color coded as indicated. RNAU6B was used as endogenous control for normalization and paired-two-tailed t-tests was used to evaluate differences in expression.

**Additional file 4: Figure S3.** Functional target network validation of 9 differentially expressed miRNAs and 9 predicted target mRNAs. The comparison was done between atrial myocardial tissue of CHD patients before CBP (n=10) and after CPB (n=10). RNAU6B and GAPDH were used as an endogenous controls for normalization of miRNA and mRNA, respectively. Paired-two-tailed t-tests and ± standard deviation (STDV) were used to evaluate differences in expression.

**Additional file 5.** 1) Information letter to patients and parents, 2) consent form for anonymous publication, participation & storage of medical data and 3) tissue sample form.

## Abbreviations

AF: atrial fibrillation
ASD: atrial septal defect
CDKN1A: cyclin dependent kinase inhibitor 1A
CHD: congenital heart defect
c-Myc: v-Myc avin myelocytomastosis viral oncogene homologue
CPB: cardiopulmonary bypass
DMSO: dimethyl sulfoxide
d-TGA: dextro-position of the great arteries
Elk1: eTS transcription factor
ESR1: estrogen receptor 1
ETS1: eTS proto-oncogene 1
FDA: false discovery rate
GAPDH: glyceraldehyde 3-phosphate dehydrogenase
HLHS: hypoplastic left heart syndrome
HNF4A: hepatocyte nuclear factor 4 alpha
KEGG: Kyoto encyclopedia of genes and genomes
KRAS: KRAS proto-oncogene, GTPase
LAA: left atrial appendages
MGMT: O-6-methylguanine-DNA methyltransferase
miEAA: miRNA enrichment analysis and annotation
miRNA: microRNA
PRBCs: packed red blood cells
PTEN: phosphatase and tensin homolog
RAA: right atrial appendages
RMVD: rheumatic mitral valve disease
RT-qPCR: quantitative reverse transcription-polymerase chain reaction
RVOT: right ventricular outflow tract
SERCA2a: ATPase sarcoplasmic/endoplasmic reticulum Ca2 + transporting 2
SOD2: superoxide dismutase 2
TAPVC: total anomalous pulmonary venous connection
TGS: total gene signals
TOF: tetralogy of fallot
VSD: ventricular septal defect
