# Supplementary material for: Differential expression of microRNAs following cardiopulmonary bypass in children with congenital heart diseases
Source: J Transl Med. 2017 May 30;15:117. doi: 10.1186/s12967-017-1213-9 (PMC5450060; doi:10.1186/s12967-017-1213-9)
Supplement: Supplementary file 1 — Additional file 1: Table S1. RT-qPCR primer sequences used in the study. Table S2. MiRNA associated pathways in the atrial myocardial tissue of patients with CHD after CPB (n=3) compared to before CPB (n=3): KEGG Pathways with predicted interaction enrichments for deregulated miRNAs by microarray (P value < 0.05). [file 12967_2017_1213_MOESM1_ESM.docx]

**Supplemental Table 1:** miScript and QuantiTect Primer Assays used in the study.

| **miRNA** | **miScript Assays Sequence (Qiagen, Hilden, Germany)** |
| --- | --- |
| hsa-miR-328-5p | GGGGGGGCAGGAGGGGCUCAGGG |
| hsa-miR-4750-5p | CUCGGGCGGAGGUGGUUGAGUG |
| hsa-miR-210-5p | AGCCCCUGCCCACCGCACACUG |
| hsa-miR-423-3p | AGCUCGGUCUGAGGCCCCUCAGU |
| hsa-miR-564 | AGGCACGGUGUCAGCAGGC |
| hsa-miR-770-5p | UCCAGUACCACGUGUCAGGGCCA |
| hsa-miR-874-5p | CUGCCCUGGCCCGAGGGACCGA |
| hsa-miR-93-5p | CAAAGUGCUGUUCGUGCAGGUAG |
| hsa-miR-744-5p | UGCGGGGCUAGGGCUAACAGCA |
| hsa-miR-648 | AAGUGUGCAGGGCACUGGU |
| hsa-miR-193b-3p | AACUGGCCCUCAAAGUCCCGCU |
| hsa-miR-212-3p | UAACAGUCUCCAGUCACGGCC |
| hsa-miR-143-3p | UGAGAUGAAGCACUGUAGCUC |
| hsa-miR-222-3p | AGCUACAUCUGGCUACUGGGU |
| hsa-miR-423-3p | AGCUCGGUCUGAGGCCCCUCAGU |
| hsa-miR-766-3p | ACUCCAGCCCCACAGCCUCAGC |
|  |  |
| **miRNA** | QuantiTect Primer Assays (Qiagen, Hilden, Germany) |
| Hs_CDKN1A | QT00062090 |
| Hs_MYC_1 | QT00035406 |
| Hs_PTEN_1 | QT00086933 |
| Hs_ESR1 | QT00044492 |
| Hs_ETS1_1 | QT00049133 |
| Hs_SOD2_1 | QT01008693 |
| Hs_MGMT_1 | QT01004416 |
| Hs_KRAS_1 | QT00083622 |
| Hs_HNF4A | QT00019411 |
| Hs_GAPDH | QT00079247 |

**Supplemental Table 2:** MiRNA associated pathways in the atrial myocardial tissue of patients with CHD after CPB (n=3) compared to before CPB (n=3): KEGG Pathways with predicted interaction enrichments for deregulated miRNAs by microarray (P value < 0.05)

| **KEGG pathway** | ***P-*value, FDR corrected** | **#genes** | **#miRNAs** |
| --- | --- | --- | --- |
| Hippo signaling pathway | 1,11E+05 | 114 | 70 |
| Glutamatergic synapse | 2,62E+05 | 88 | 66 |
| Pathways in cancer | 2,62E+05 | 286 | 79 |
| Adrenergic signaling in cardiomyocytes | 1,20E+06 | 112 | 71 |
| Phosphatidylinositol signaling system | 1,23E+06 | 66 | 63 |
| Morphine addiction | 3,20E+06 | 72 | 62 |
| TGF-beta signaling pathway | 3,29E+06 | 64 | 53 |
| cGMP-PKG signaling pathway | 3,29E+06 | 128 | 74 |
| N-Glycan biosynthesis | 0,0001 | 35 | 37 |
| Proteoglycans in cancer | 0,0001 | 142 | 71 |
| Chronic myeloid leukemia | 0,0003 | 58 | 56 |
| Estrogen signaling pathway | 0,0003 | 74 | 65 |
| Endocytosis | 0,0003 | 147 | 70 |
| Glioma | 0,0005 | 50 | 58 |
| Retrograde endocannabinoid signaling | 0,0007 | 78 | 66 |
| Axon guidance | 0,0009 | 95 | 70 |
| Colorectal cancer | 0,0010 | 49 | 53 |
| FoxO signaling pathway | 0,0010 | 98 | 72 |
| Mucin type O-Glycan biosynthesis | 0,0018 | 22 | 36 |
| Renal cell carcinoma | 0,0018 | 51 | 49 |
| Long-term depression | 0,0027 | 47 | 54 |
| ErbB signaling pathway | 0,0027 | 67 | 62 |
| Oxytocin signaling pathway | 0,0029 | 115 | 72 |
| MAPK signaling pathway | 0,0030 | 179 | 77 |
| Glycosaminoglycan biosynthesis - chondroitin sulfate / dermatan sulfate | 0,0032 | 15 | 24 |
| Adherens junction | 0,0032 | 56 | 58 |
| HTLV-I infection | 0,0032 | 182 | 74 |
| Melanoma | 0,0035 | 54 | 56 |
| Thyroid hormone signaling pathway | 0,0035 | 86 | 67 |
| Ras signaling pathway | 0,0035 | 155 | 76 |
| Prostate cancer | 0,0041 | 67 | 66 |
| Focal adhesion | 0,0044 | 146 | 74 |
| Circadian rhythm | 0,0046 | 26 | 38 |
| Dopaminergic synapse | 0,0050 | 96 | 73 |
| Small cell lung cancer | 0,0060 | 65 | 57 |
| Endocrine and other factor-regulated calcium reabsorption | 0,0061 | 35 | 54 |
| Amphetamine addiction | 0,0065 | 48 | 61 |
| Bacterial invasion of epithelial cells | 0,0074 | 58 | 55 |
| Ubiquitin mediated proteolysis | 0,0079 | 101 | 65 |
| Hepatitis B | 0,0085 | 100 | 69 |
| Thyroid hormone synthesis | 0,0086 | 51 | 51 |
| GABAergic synapse | 0,0089 | 67 | 62 |
| Gap junction | 0,0106 | 65 | 61 |
| Circadian entrainment | 0,0107 | 73 | 67 |
| Sphingolipid signaling pathway | 0,0145 | 85 | 64 |
| Choline metabolism in cancer | 0,0151 | 74 | 69 |
| Insulin secretion | 0,0154 | 64 | 57 |
| Dilated cardiomyopathy | 0,0154 | 65 | 57 |
| Arrhythmogenic right ventricular cardiomyopathy (ARVC) | 0,0174 | 53 | 54 |
| Calcium signaling pathway | 0,0174 | 124 | 71 |
| GnRH signaling pathway | 0,0178 | 67 | 61 |
| Neurotrophin signaling pathway | 0,0178 | 85 | 69 |
| AMPK signaling pathway | 0,0189 | 86 | 67 |
| cAMP signaling pathway | 0,0190 | 137 | 71 |
| Vascular smooth muscle contraction | 0,0196 | 83 | 65 |
| Endometrial cancer | 0,0201 | 39 | 57 |
| Pancreatic cancer | 0,0211 | 51 | 57 |
| TNF signaling pathway | 0,0229 | 76 | 63 |
| Nicotine addiction | 0,0242 | 27 | 55 |
| Rap1 signaling pathway | 0,0243 | 143 | 70 |
| Long-term potentiation | 0,0248 | 50 | 59 |
| Cell adhesion molecules (CAMs) | 0,0248 | 98 | 68 |
| Regulation of actin cytoskeleton | 0,0248 | 144 | 73 |
| Platelet activation | 0,0297 | 88 | 62 |
| Signaling pathways regulating pluripotency of stem cells | 0,0334 | 97 | 69 |
| Inositol phosphate metabolism | 0,0369 | 44 | 49 |
| Inflammatory mediator regulation of TRP channels | 0,0399 | 70 | 59 |
| Shigellosis | 0,0458 | 46 | 49 |
| Cocaine addiction | 0,0479 | 33 | 51 |
| Cholinergic synapse | 0,0479 | 80 | 61 |
