# Supplementary figures and images for: Differential expression of microRNAs following cardiopulmonary bypass in children with congenital heart diseases
Source: J Transl Med. 2017 May 30;15:117. doi: 10.1186/s12967-017-1213-9 (PMC5450060; doi:10.1186/s12967-017-1213-9)

## Slide 1
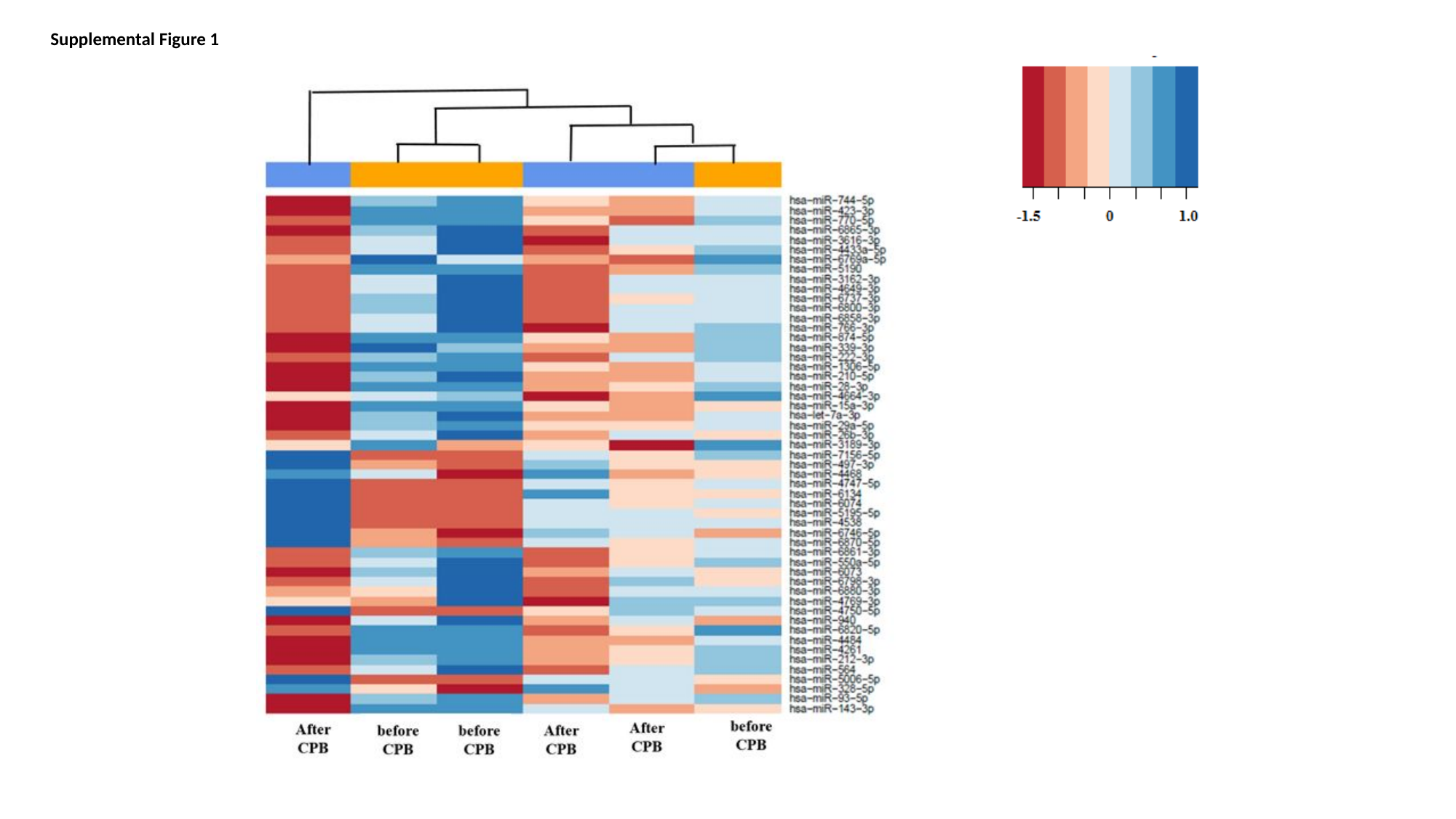

Supplemental Figure 1

Supplement: Supplementary file 2 — Additional file 2: Figure S1. Unsupervised hierarchical clustering (Euclidian distance, complete linkage) of 3 patients with 6 samples including 3 samples before and 3 samples after CPB. The clustering was done based on the expression of the highest variance miRNAs as determined by RT-qPCR. MiRNAs with high expression are shown in blue and miRNAs with low expression in red. The blue and orange lines indicate the two main clusters of samples. [file 12967_2017_1213_MOESM2_ESM.pptx]

## Slide 1
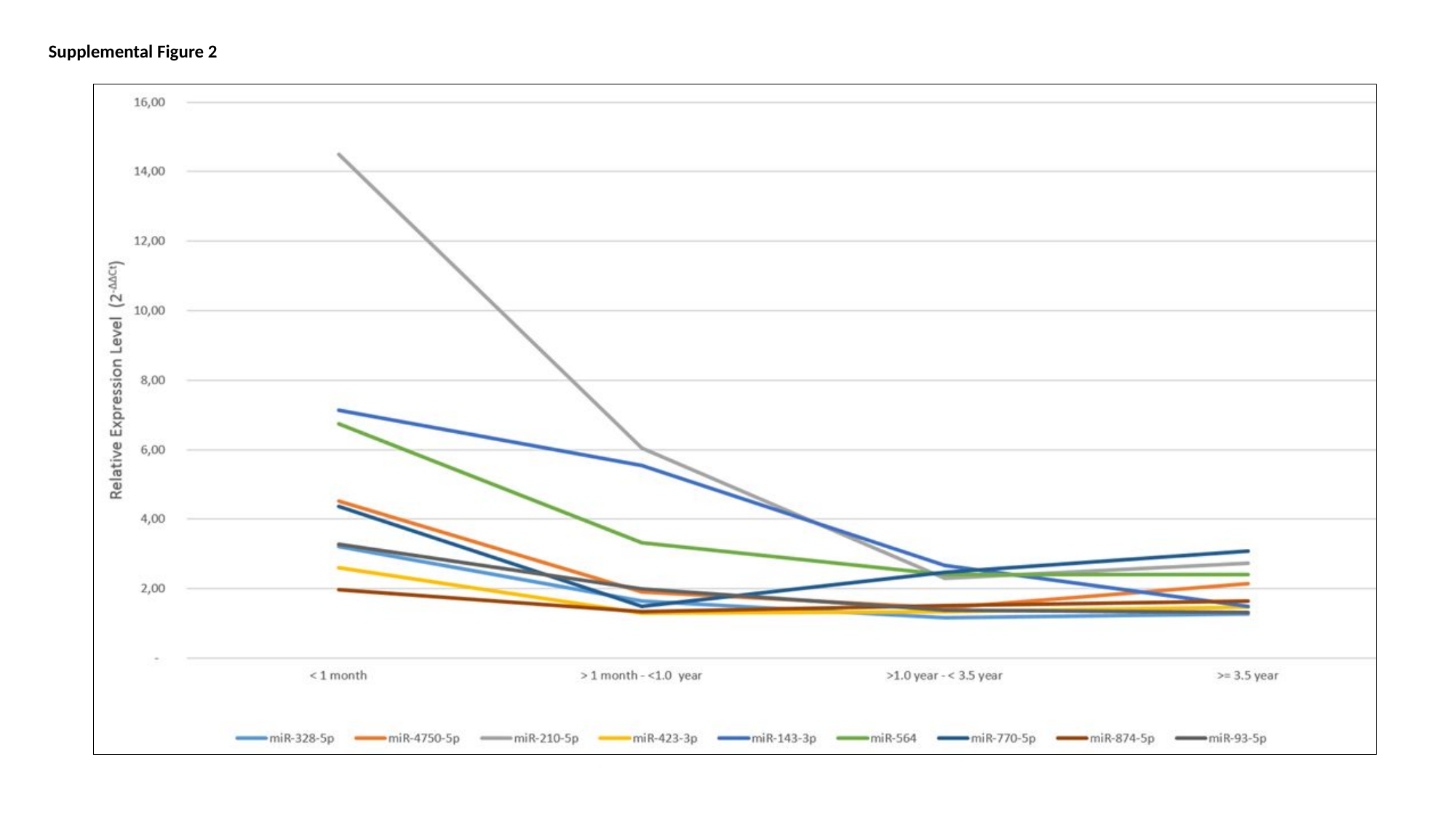

Supplemental Figure 2

Supplement: Supplementary file 3 — Additional file 3: Figure S2. Influence of age on the expression of 9 differentially expressed miRNA as determined by RT-qPCR. The relative expression level of 2-ΔΔCt is indicated on the y-axis and the patient age at the time of surgery on the x-axis. The different miRNAs are color coded as indicated. RNAU6B was used as endogenous control for normalization and paired-two-tailed t-tests was used to evaluate differences in expression. [file 12967_2017_1213_MOESM3_ESM.pptx]

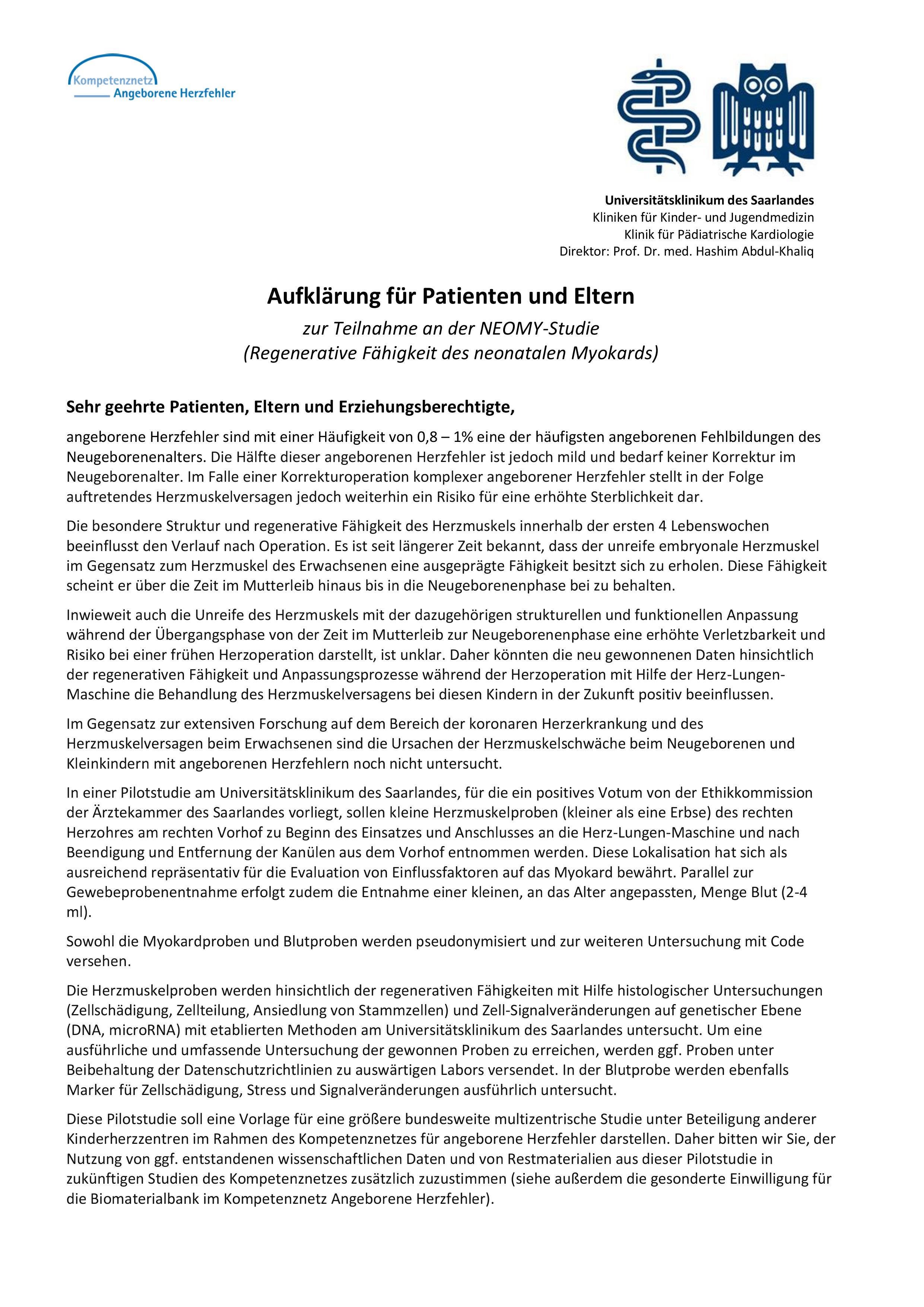


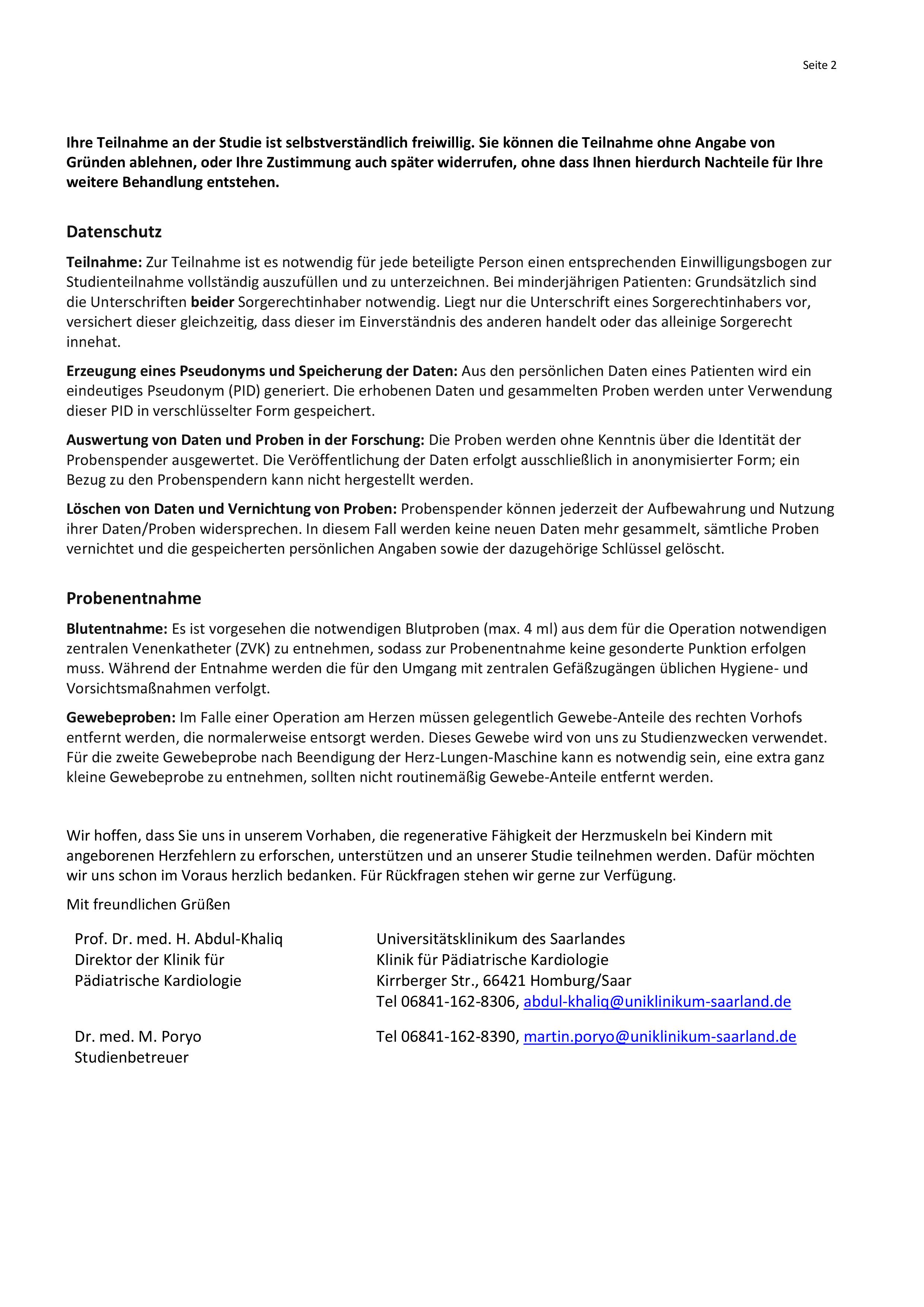


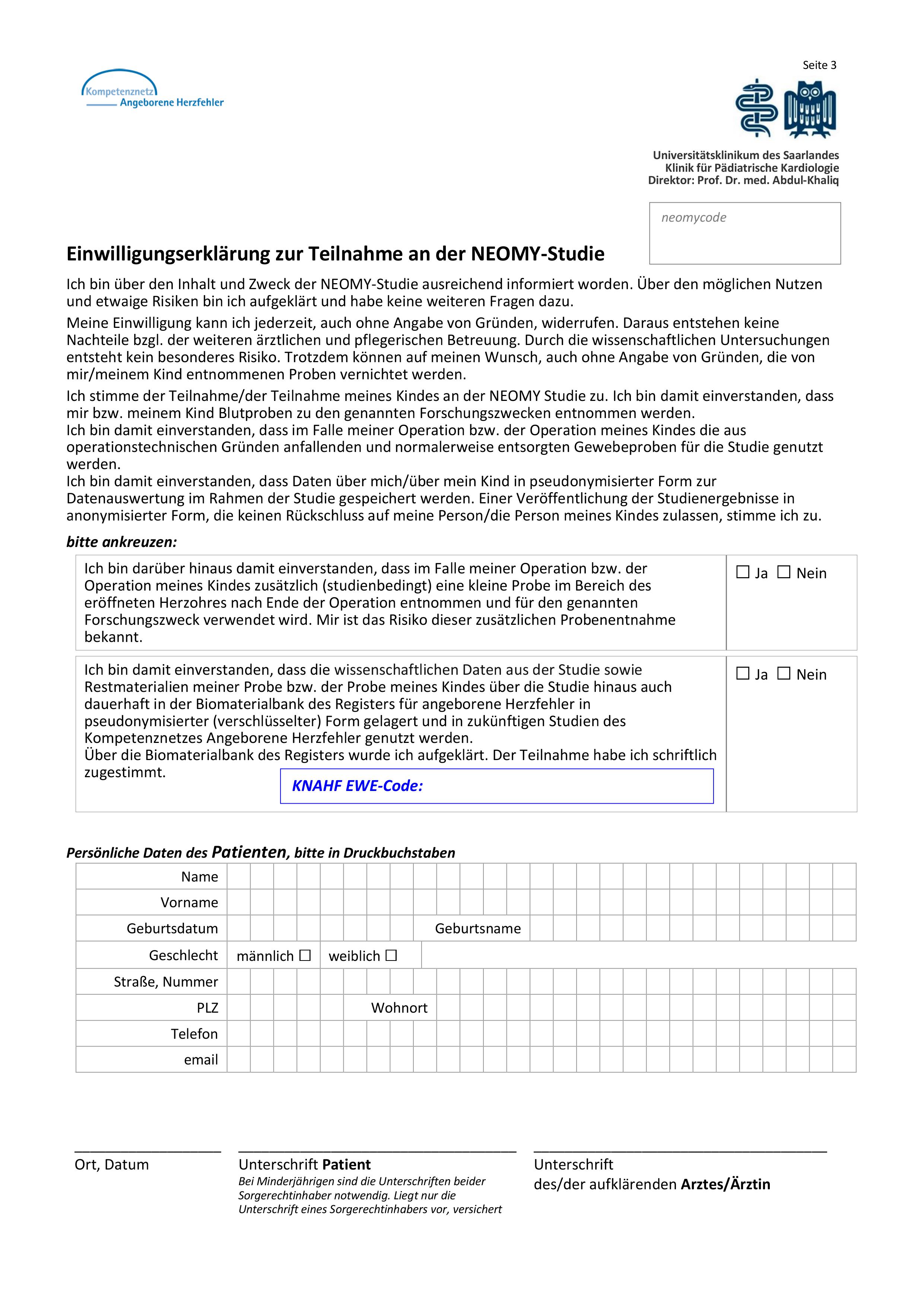


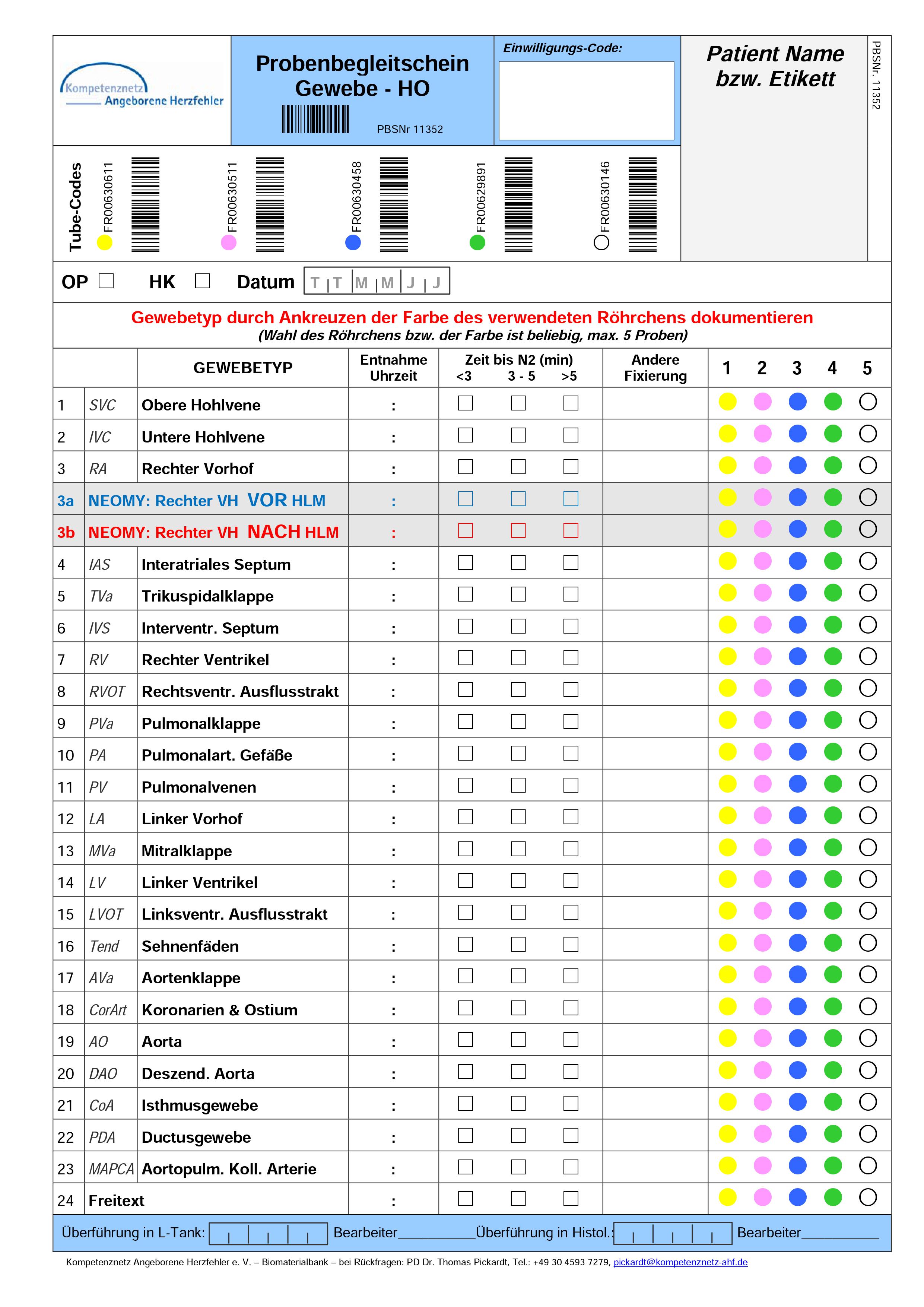

Supplement: Supplementary file 5 — Additional file 5. 1) Information letter to patients and parents, 2) consent form for anonymous publication, participation & storage of medical data and 3) tissue sample form. [file 12967_2017_1213_MOESM5_ESM.docx]
