# Supplementary material for: Differential expression of microRNAs following cardiopulmonary bypass in children with congenital heart diseases
Source: J Transl Med. 2017 May 30;15:117. doi: 10.1186/s12967-017-1213-9 (PMC5450060; doi:10.1186/s12967-017-1213-9)
Supplement: Supplementary file 4 — Additional file 4: Figure S3. Functional target network validation of 9 differentially expressed miRNAs and 9 predicted target mRNAs. The comparison was done between atrial myocardial tissue of CHD patients before CBP (n=10) and after CPB (n=10). RNAU6B and GAPDH were used as an endogenous controls for normalization of miRNA and mRNA, respectively. Paired-two-tailed t-tests and ± standard deviation (STDV) were used to evaluate differences in expression. [file 12967_2017_1213_MOESM4_ESM.pptx]

## Slide 1
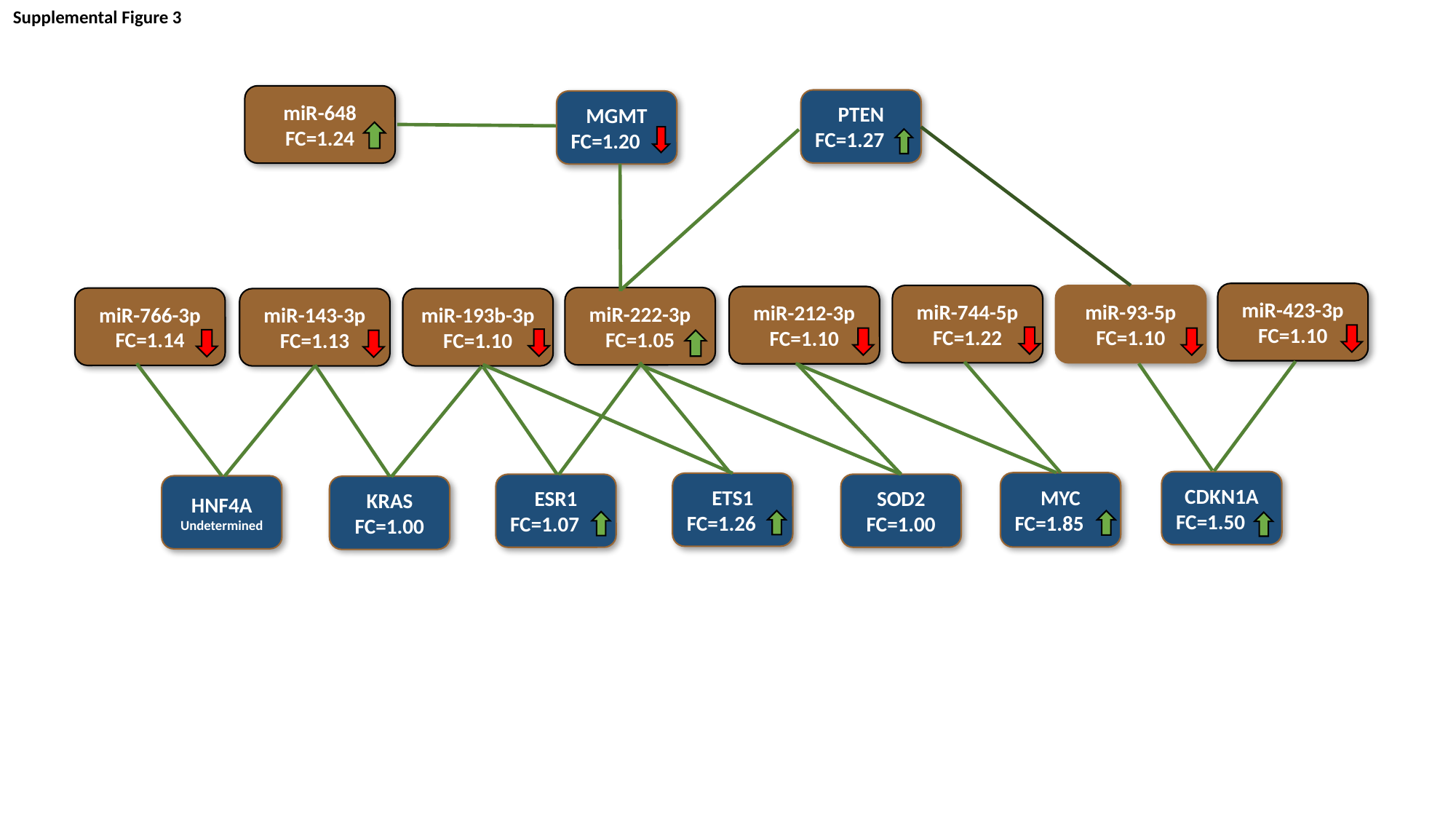

Supplemental Figure 3
miR-648
FC=1.24
PTEN
FC=1.27
MGMT
FC=1.20
miR-423-3p
FC=1.10
miR-744-5p
FC=1.22
miR-93-5p
FC=1.10
miR-212-3p
FC=1.10
miR-222-3p
FC=1.05
miR-766-3p
FC=1.14
miR-143-3p
FC=1.13
miR-193b-3p
FC=1.10
CDKN1A
FC=1.50
MYC
FC=1.85
ETS1
FC=1.26
SOD2
FC=1.00
ESR1
FC=1.07
HNF4A
Undetermined
KRAS
FC=1.00
